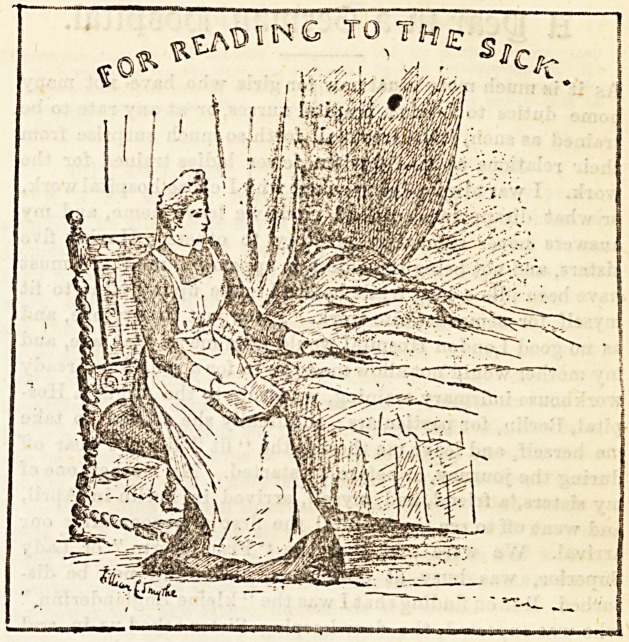# The Hospital Nursing Supplement

**Published:** 1891-06-06

**Authors:** 


### "The Hospital" Nursing Mirror.

Being the Extra Nursing Supplement of "The Hospital" Newspaper.

Contributions for this Supplement should be addressed to the Editor, The Hospital, 140, Strand, London, W.C., and should have the word "Nursing" plainly written in left-hand top corner of the envelope.

### En Passant.

NOTTINGHAM NURSES.—The members of the Nottingham and Notts Nursing Association have been overpowered by work during the last two months, and dozens of cases have been refused daily. The report of last year's work is good; the nurses attend both rich and poor, and several letters published prove how gratitude repays the nurses for their services. Miss Forrest, the Lady Superintendent, is specially thanked by the Committee for her skill and constant interest in the management of the institution.

THE DEWSBURY INQUEST.—An inquest was lately held in Dewsbury on the body of a child scalded to death, and some hints of infirmary mismanagement led to an enquiry, which has ended in the issue of the following report: "'That this Committee having investigated the charge brought against the House Surgeon, Matron, and Nurse, find that the House Surgeon was of opinion that the child was too young to be admitted, and would be better at home under the care of his mother. He was also under the impression that children so young were not admitted. The Matron and Nurse stated that they had said that they thought the doctor would not admit a child so young.' The committee consider they had made an error of judgment, and gave instructions that in future all accidents should be admitted at any time."

UPPER NORWOOD DISTRICT NURSING. — The above work is largely supported by members of Central Hill Chapel, the minister being Mr. Tipple, also Chairman of the Committee. He is ably helped by Mrs. Pritchard, the Secretary, and Mrs. Canhand, Hon. Secretary, and a committee of six ladies who help the midwifery nurse in her work. For the past year, ending with May 6th, there were 48 midwifery patients; 36 nights nurse stayed with patients; and 1,368 sick visits were paid. One midwife, who has now resigned her post for better work, has done all, taking all cases non-infectious that have come under her notice, only calling in medical aid for two midwifery cases, and on one occasion a committee lady, who has left through ill-health, volunteered one night up to help nurse.

SHORT ITEMS.—Her Excellency the Countess of Zetland has sent a cheque for £3 to the Royal National Pension Fund for Nurses.—A patient named Holmes, in an attack of delirium, severely wounded one of the nurses of York County Hospital with a pen-knife.—The Queen of Sweden, on her nameday, gave 1,000 kr. to the Sick Nurses' Pension Fund.—The late Lady Verney, sister of Florence Nightingale, wrote a series of stories called "The Grey Pool," which will shortly be published.—The first examination of asylum attendants has been held by the Medico-Psychological Society. There were numerous candidates, and those that pass will be presented with certificates.—The district nurses of Chester have done so well under Miss Vickers, that they are to be moved from the Deaconess Institute into a home of their own.—On May 13th, at Teddington Church, Mr. G. Q. Roberts, Secretary of the London Hospital, was married to Miss M. L. Waters, for many years Assistant Matron at the same institution.

NURSES' MISSIONARY ASSOCIATION.—The report of the work done by this association during 1890 shows how truly many nurses enter into the spirit of their profession, and heed every call for help. The hospital at Mahanora has received £10, North China Mission, £12; and the South African Mission, £20. All this money was derived from the shilling annual subscriptions of the members, most of whom are nurses. A proper balance-sheet is published, and the association seems to be excellently managed. The Secretary is Miss D. M. Image, 53, Gloucester Street, S.W.

ANOTHER HOME.—We are glad to hear that Miss Maud Lobb has started a Convalescent Hospital for Children at Hillside, Lambourne, Essex. Miss Lobb is the daughter of the late Dr. Lobb, of Hampstead, and for some time has been working in Australia, where she held the post of Matron at the charming Children's Hospital in Sydney. Miss Lobb means to take children with open sores, and just those cases which are excluded from ordinary homes. There are to be very few rules, and the youngsters are to be as merry and free as circumstances will admit. The charge is to be 7s. 6d. a-week at first, though Miss Lobb hopes to reduce this later on.

TO WOULD-BE NURSES.—The enquiry into the insanitary condition of St. Bartholomew's Hospital has brought out very fully the folly of the present rush on nursing. In the report of Dr. Sedgwick Saunders it is stated: "Many of the nursing staff probationers were young, immature women, unaccustomed to hard menial work, who were translated from ward to ward in which infectious cases were alone treated before their systems had become acclimatised to disease, or their bodies inured to the drudgery they must submit to in passing through their training. He thought the age of admission of these nurses was too low. In these circumstances, well-born ladies were more liable to break down, and more susceptible to infection than others." In fact, if ladies wish to nurse the sick and dying they must understand that they do so at the risk of their lives, and they must not lightly enter on a path which may lead them to the grave.

THE BELFAST SOCIETY.—Belfast can boast one of the best district nursing societies in Ireland; a society which nursed 809 poor patients last year. The Mayor very properly presided at the annual meeting held on May 22nd, when there was a large attendance including many ladies. Mrs. Higgins, submitted the financial statement, which showed a balance in the bank of £959 3s. 3d., from which however, £150 had been invested since the compilation of the statement and report. Notwithstanding this investment, however, the balance was still larger than it had been last year. The Moderator, in moving the adoption of the report and statement of accounts, said he had great pleasure in moving this charming report, for it was beautifully written. It told them about the works of women who were filled with love to others, and who were anxious to do good. Mr. Mitchell moved—"That this meeting acknowledges with thankfulness the progress, under the Divine blessing, the society has made during the past year; and, in order to maintain in full efficiency the nurses now employed, earnestly commends the society to the sympathy and help of the many friends of the poor in Belfast and neighbourhood." This was carried with applause, and a very satisfactory meeting was concluded with prayer.

### Lectures on Surgical Ward Work and Nursing.

By Alexander Miles, M.B. (Edin.), C.M., F.R.C.S.E.

### Lecture XXVI.

### Box Splint.

This splint, which is often associated with the name of the Edinburgh school, is largely used there in the treatment of all fractures of the bones of the leg, as well as in other conditions in which it is necessary to control the movements of this part of the limb. It has the great advantage of being readily and quickly made, all the requisites beirg procurable anywhere.

Materials Required.—(1) Two wooden splints; (2) a small sheet; (3) a towel; (4) some cotton wool, antiseptic if possible; (5) four "nests" of cotton wool; (6) a cotton bandage for slip knots; (7) a domette bandage for foot; (8) scissors, safety pins, &c.

Method of Preparing the Materials.—The *splints* should be of strong wood, measuring four inches in breadth and extending from above the knee to three or four inches beyond the sole. The *sheet* having been folded to correspond with the length of the splints, one of the latter is laid on each end of the sheet and rolled up in it. Three sides of a box are thus formed, and the splint should be fitted on to the sound limb (allowing for padding) to avoid disturbing the injured part unnecessarily. If it be found that the space between the two sides is not accurate, only one end should be unrolled, and by folding in or letting out the sheet, the proper size will be obtained. If you undo both ends, the chances are the error will be in the opposite direction next time. The *wool* is used to pad the whole of the inner surface of the box, and with it small round rings, or "nests," are made to place over the condyles of the femur at the knee and the malleoli at the ankle to avoid undue pressure on these prominences. The hollow behind the heel, *i.e.*, over the tendo achilles, and the space behind the knee (popliteal space), also require special support. The *towel* is then folded so as to cover in the front of the leg, forming, so to speak, the lid of the box. The *slip knots* are made thus: An ordinary six-yard cotton bandage is run out and divided into three equal parts. Each part doubled upon itself constitutes a "slip knot."

Method of Applying the Splint.—This, of course, includes the setting of the fracture, which will be done by the surgeon, and the nurse's duty is to assist him. The bones having been put into proper position, and being held there, the splint is slipped under the limb and the sides of the box are then folded up, the pads carefully adjusted, so that the pressure is properly distributed, and the folded towel is laid on the front of the leg. While the whole apparatus is fixed by the assistant the three slip knots are passed under the limb from without inward so that the two free ends lie towards the outside. One is placed at the middle, one just above the ankle, and the other just below the knee. The middle one should be tied first. This is done by passing one free end through the loop of the double end and pulling tight, then fixing by means of a reef bow which should be placed over the outer splint. The other two are fixed in the same way. The *domette bandage* is then applied round the foot and lower part of the leg to keep the foot at right angles with the leg, to correct any twisting of the foot that may exist, and to support the part generally.

Advantages of the Box Splint.—(1) Its simplicity. (2) Material always available. (3) Its general applicability. (4) The limb can be examined without removing the splint, and thus rest maintained.

### Gooch Splint.

Many fractures are treated by means of Gooch splints, and you should know what materials are required in this process, although it would be beyond our present purpose to detail the methods of making and applying these in individual injuries.

Materials Required.—(1) Gooch splint material, (2) saw, (3) strong knife, (4) wool for padding, (5) bandages, (6) sling, (7) scissors, safety pins, &c.

Sand bags are very useful for securing and maintaining rest. They can be placed alongside broken limbs, and so act as a temporary splint. If a patient complain of backache while being treated with extension for hip disease, this can often be relieved by placing a draw-sheet over the chest and abdomen, and fixing it by a sand-bag on each side moulded into the body of the patient. The sand must be very fine and *perfectly dry*, and should fill the bag only about two-thirds full, as if quite full it does not mould so readily to the parts. The bag should be made of some very close material, such as bed-ticking.

### Thomas' Splints.

These appliances, named after the late Mr. Thomas, of Liverpool, who introduced them, are very largely used in the treatment of joint diseases.

(1) Thomas' Knee Splint consists of two parallel iron bars surmounted by an oval padded metal ring, which is formed to fit into the perineum on the affected side. The lower end also consists of an oval ring, which rests on the ground, but beyond the level of the patient's foot. In this way the patient really sits on the upper end, with the diseased leg hanging in the splint. A patten is fixed to the boot of the opposite side to equalise the length of the limbs. The limb is bandaged to the splint above and below the knee, and a brace is passed from the upper ring in front, over the opposite shoulder, and fixed to the upper ring again behind.

(2) Thomas' Hip Splint.—This consists of a long bar of malleable iron fitted with three transverse pieces or crescent wings, which grasp respectively the chest, the thigh, and the calf. The splint is bandaged to the leg and thigh, and fixed to the body either by a strap with buckle, or by a roll of bandage.

### The Queen at Derby.

Dear Mr. Editor. — No doubt you will have received a full account of our Queen's visit to Derby, where she laid on Thursday last the foundation stone of the New Infirmary; but perhaps a letter from one of the nurses of how we enjoyed the day, may interest other nurses, so if you can will you kindly insert this in your valuable and interesting paper? At the early hour of 6.30 a.m. we night nurses had our dinner, and as soon as possible went to bed, but not to sleep, for the streets were so noisy, bells ringing, bands playing, and children screaming. We do not sleep now in the old Infirmary, but in a private house near. We got up at 2.45, had a meal, and met our Matron in the Central Hall at 3.45 p.m. I must say we did look nice, like bright new pins. Seven head nurses in navy blue stuff dresses, new linen hemstitched aprons, white muslin "Sister Dora" caps; and the probationers in their narrow mauve striped cotton dresses, white aprons and "Nightingale" caps. As we have much outdoor work to do now we wear warm scarlet cloaks; these of course we put on when we took our places on the uncovered stand to wait the Queen's arrival. There was plenty to amuse us, and often we wondered would the Queen really come herself. Oh! how eagerly we looked as the procession came in sight. Now, instead of "Will she come?" our united cry was "She has come." There she sat, looking well, but quite moved as the bursts of welcome reached her from all sides. After the procession had passed on its way to the Town Hall, we moved and took our places in the marquee; here, through the great kindness of our Committee, we were given the first central row of chairs. The waiting did not seem long; soon we heard the flourish of trumpets, and the strains of the National Anthem which told us Her Majesty had arrived. After the prayers for God's blessing on the new work, in almost solemn silence the foundation stone was laid in its place, by the Queen's own hand. Too soon she passed into the reception room, where the presentation took place. Our Matron, Miss M. Pratt, is absent through illness, and sorry we all were not to have had her among us, and I know she is sorry also not to have been with us. The Assistant Matron, Miss C. Williamson, who is doing matron's duties, was presented in her name; to her we owe a good part of the happy arrangements of the day. After the procession had started on its return to the Midland Railway Station we went and had a look at the school children, over two thousand on stands along the drive, who at intervals had been singing "God Save the Queen" and "Auld Lang Syne," varied by bursts of ringing cheers, as only children can cheer.

In the evening about fifteen of us drove round the town to see the illuminations, which were really very fine; and the next night another party of us went. Was it fine? you ask. Alas, no—though it might have been worse; small rain had fallen all day, but, just as the Queen came by, a faint gleam of sunlight broke out—it soon passed.

Her Majesty was most kind and thoughtful in allowing her carriage to remain open, and she used no umbrella. All the wards looked so extra bright and pretty; the head nurses had taken the greatest trouble for weeks past, making new table covers, &c.; the plants and cut flowers were splendid. Every patient who could with safety be got up went to the windows in front of the old building, and there saw the procession pass twice. The servants were on the small balcony, and had a good view. Every nurse saw the Queen. Those who minded the wards when the procession passed first were relieved by those who had seen it, and they came and joined us in the marquee. I am sure never did nurses spend a happier day, and none could have been more thought of. Dr. Ogle added much to our pleasure by presenting us each with a commemoration medal, which we wore on our dresses fastened by a piece of blue ribbon. The day at last came to an end for the day people, and we night nurses went on duty, tired but very happy. I think after all we had the best of it, for we could continue talking over the great event. Fancy, we did not have one accident in; not even a casualty. God bless our most Gracious Queen! and may God bless the Royal Infirmary is the prayer of every nurse here. Hoping you will forgive such a long letter,

F. W. W.

### Appointments.

[It is requested that successful candidates will send a copy of their applications and testimonials, with date of election, to The Editor, The Lodge, Porchester Square, W.]

Bromley Cottage Hospital.—Miss Heather-Bigg, who trained at Guy's, has been appointed Matron at Bromley, one of the largest cottage hospitals in England.

London Hospital.—Miss H. Disney, Miss J. Rodgers, and Miss F. C. Loirane have been appointed Sisters. All trained at the London.

Poplar Hospital.—Miss Vacher, who trained at the London, and has for some years acted as Matron of the Eccles and Patricroft Hospital, has been appointed Matron at Poplar.

Peterborough Infirmary.—Miss Emma Going has been unanimously elected Matron of the Peterborough Infirmary and Dispensary. Miss Going was at one time Matron at Truro, and also at the Carlisle Fever Hospital; lately she has acted as Matron of the Middlesborough Fever Hospital. Her testimonials are excellent, and we wish her all success at her new post.

### WORK IN IDLENESS.

We are all much happier when we are work. Nothing is more wretched than being obliged to rest the body, while the mind is craving for employment, and yet in some cases this very rest, so hateful, is the means of our cure.

It is absolutely necessary for many a sick person to keep every limb and muscle perfectly quiet, and if the mind can be a blank almost, the quicker and surer will be the return to health. At the same time there are a good many who are all the better for being employed; a little occupation, a few kind thoughts or words to others in their ailments, take them out of themselves and pass their idle time pleasantly.

For instance, we are feeling a great deal better after a week of pain and discomfort, and we would like to get up and walk about, and think we should soon be all right if we could do so, instead of which we are ordered to lie still and be quiet a little longer.

Do we make the best of it? Unfortunately no. We are impatient and restless, grumble at doctor, nurses, and friends, get feverish, have a splitting headache, and make up our minds nobody was ever so ill-used as we are. Now this may be termed in common phrase "a piece of work," but not of the sort we ought to do. Let me tell you, dear friend, what the work is that you have had put into your hands, to conquer yourself. Solomon, the wisest man that ever lived, says, "He that ruleth himself is better than he that taketh a city," for the one brings peace and calm, and the other noise, cruelty, and misery.

Resolve not to think of your troubles, but count up your blessings, what a number you have though you *must* do as you are bid at present, and that goes against the grain sadly. It is good for grown people as well as children to have an obedient spirit, not a blind giving yourself up to do all that is told you, good or bad, without questioning, but to encourage a teachable spirit, one ready to receive instruction, what was called by old people a "condescending temper." We all know the difference between a person ready to oblige and one who, though he says "Yes," always adds a "but." "But," implies so much, such a determination to do a kindness so long as it is not "ill convenient."

But we will not dwell on our neighbours faults, hardly on our own just now, since we are weak and out of sorts, so we will simply make up our minds that whether in health or sickness we will try to make our lives as near like our dear Master's as we can, of whom we are told that "Christ pleased not Himself." We must work His work while it is day lest the night cometh when no man can work. No *great* thing is expected of us, indeed it is wonderful how little God requires if we have His spirit in our hearts.

"The daily round the common task
Will furnish all we need to ask,
Room to deny ourselves, a road
To lead us daily nearer God."

### A Year in a German Hospital.

As it is much more usual now for girls who have not many home duties to become hospital nurses, or at any rate to be trained as such, it does not call forth so much surprise from their relations as it did when fewer ladies trained for the work. I was always being asked why I chose hospital work, or what disappointment had made we leave home, and my answers never seemed satisfactory to others. Having five sisters, and not being the eldest, it appears to me that I must have been idle a long while before I made up my mind to fit myself for some definite work. I was just twenty-one, and as no good London Hospital would take me at that age, and my mother would not allow me to go in for a rough-and-ready workhouse infirmary training, we wrote to the Augusta Hospital, Berlin, for particulars; and finally she settled to take me herself, and leave me there if the "fit" did not wear off during the journey, or before we started. My mother, one of my sisters, a friend, and myself, arrived in Berlin in April, and went off to see the hospital the first afternoon after our arrival. We were told that the "Frau Oberin," or Lady Superior, was busy at operations, and could not be disturbed. But on finding that I was the "kleine Engländerinn," who was expected, the door-keeping Sister asked us in, and fetched the Oberin during an interval in her work. I think the good lady regretted having agreed to take me, when she saw how young I looked, and how small I was; I know I looked frivolous—indeed, she afterwards told me she had thought so. However, she listened to all that my mother had to say, and settled that I should come in two days. We were then shown over all the wards by a Sister who could not speak English at all.

The hospital was founded during the Franco-German War of 1870-71, to be worked by ladies of the higher classes who wished to give as much time as they could spare from other duties to nurse the sick and wounded. It was chiefly supported by the Empress Augusta, after whom it was named, and by whom the rules were drawn up. She kept the approval of the appointments of doctors, sisters, and pupils entirely in her own hands after they had been chosen by a committee of ladies and gentlemen to whom all questions were referred, and the institution was under her personal supervision during her life. At the end of the war, the buildings, which at first were quite temporary—mere tents and barracks—were rebuilt by degrees. The hospital stands in a lovely piece of open ground near the "Invaliden Garten" in the "Scharnhorst Strasse." During the war the help of all, even if they could only offer certain hours of the day or night, was eagerly accepted; but when that was over it became necessary for the permanent good of the hospital to make rules and restrictions. The Empress wished it to be worked by ladies who were willing to do all kinds of work, housework as well as nursing, and who, for the time they resided there, were to be under certain vows. Anyone who wished to train as a "Schwester" and to enter the community was to be of noble parentage on both sides of the family; she must be unmarried, must belong to the Evangelical faith, and was to promise entire submission to the Oberin. She must further be a probationer for one year, and after that period of trial she could leave, or become a fully professed "Schwester." The Empress was present at the ceremony of professing a sister, and gave her a large silver cross with an enamelled red cross in the centre, also her blue dress and cape. The profession was concluded by general rejoicing, which in the Fatherland invariably takes the form of ponderous feeding.

When I was there the whole working staff consisted of twelve sisters fully professed, two probationers, two sisters from another establishment who were training, twelve wardmaids, four trained men nurses, one "Bruder," who was learning, and four ladies who paid "pension" of £30 a-year and stayed as long as they liked. This last was the class to which I belonged, but after my first three months, the Oberin informed me I need not pay my "pension" any longer, as I did as much work as one of the sisters, and was capable of responsibility. A Dutch lady, who had been there two years, did not pay pension either. There were also six housemaids, who kept the sisters' and ladies' rooms tidy, and cleaned the passages, a carpenter, a night watchman or porter, who took in arrivals during the night, a factotum who helped at operations, and manufactured materials for dressings, and kept the surgical instruments clean and in order, an inspector, who kept the accounts, received the payments of patients, and the resident doctor's man-servant. Some women who helped in the laundry work, came in from outside by the day, otherwise all the workers lived on the premises.

In the four several buildings were 170 beds in all, a large sitting-room for the sisters, a large conference-room, a chapel, several dining halls, a mortuary, a dispensary, and quite a separate special building for diphtheria, besides the usual necessary rooms and offices attached to all hospitals. Three doctors were resident, and the two professors came every day. The doctors went round the wards twice daily, the professors once or twice a week, out-patients were seen every morning, and wardmaids, thoroughly trained, were sent out to private houses, but the sisters were never sent out. The patients were all of the paying class, and arranged according to three scales of payment, viz., 8 marks (nearly eight shillings) per day, 5 marks, and 1 mark 75 pf. Children 1 mark 25 pf. The first-class patients had private rooms, the second-class a large room between two or three, besides being fed much better in proportion than those who paid on the lowest scale.

The day arranged for my arrival came, and I must confess to feeling rather lonely when my mother left me there, and I knew that the next day she would leave Berlin. Shown into my room, I began to unpack, as seeing one's things about is the best way to feel at home. I was fetched by a sister to coffee, and when I found myself among twenty women, all in large frilled white caps and aprons, all talking German very fast, and all in rather a hurry, I confess I felt much out of place and very strange. However, they were exceedingly kind to me, and did their best to make up for my ignorance of German and their ignorance of English. I put on an apron and was taken into the children's ward directly after coffee, and there I felt at home at once. I was introduced as "Misschen," and am always called so now by them. One little girl of four years old took a great fancy to me, but I am afraid I did not at once respond, as she had a hare-lip which had been very recently operated upon, and the sight of it made me feel very sick. I liked her for welcoming me for all that. The next morning I began my duties at six o'clock, being called at half-past five by the night nurse, and having put on a short-skirted print dress with neat collar and cuffs and a large apron, I felt less out of place and strange. I had only to wash and dress some of the children and make beds, and so I got on very well. It happened to be a special kind of day; in fact, I thought at first it was a fast day of great rigour, as we had a long, dry service in the chapel, standing nearly all the time; but later on it seemed to change into a feast day, for we had a grand dinner and cake for tea.

My ward really consisted of two small wards opening on to a passage; in each ward were ten beds, and cots were added when required. One room was supposed to be for boys and the other for girls, but in practice they often invaded each other. At the end of each ward, and adjoining them, was a long glass corridor, looking over the exercise ground of the barracks, which afforded daily amusement to such children as could be out there to watch the drills, practises, and reviews. A governess came every morning for two hours, and gave lessons to all the children who were well enough; this was an excellent plan, as it gave them employment, and kept the room quieter for the very ill ones and the babies, who could then get to sleep. When I first came, ten out of twenty-five children were hip-disease cases, and there were two hare-lipped babies and one little girl; the others were ordinary, rather uninteresting cases. The pet of all was a boy of two, who looked the picture of health and happiness, with a fat, round face, fair hair, and laughing blue eyes. He was so plump and rosy that I could not believe him ill, as I saw him crawling along the floor, until I noticed that he only used his hands and body to wriggle along, his legs and feet being hardly more than sticks, much the size of those of a baby of a few months old.

He treated these limbs as playthings, shouldered them as guns, or beat the other children with them. He was an orphan, and no one could ascertain how he had become lame. The Oberin came every morning and massaged him all over; he was then seated in a hip bath of hot sand for two hours. At the end of my year he was able to walk with the help of a chair and boots with iron supports. His legs were certainly bigger, and more formed, and the muscles a little more developed. Later on we had a somewhat similar case, which was rapidly cured. A boy of ten years old, who from a fall was entirely lamed from the hips downwards, was treated with electricity, massage, and warm baths; in six months he walked with two sticks.

The sister under whom I worked was most kind in accustoming me gradually to the dressings and operations. No one could have had a greater horror of the sight of wounds or blood than I had when I came; but in a fortnight I could stand seeing and helping with any case, and liked going into the operation room.

### The Royal British Nurses' Association and its Register.

With a view to dissipating the doubts which have been artfully circulated as to the decision of the Board of Trade with reference to the application for a license by the Royal British Nurses' Association, we have been requested to publish the following letter, addressed to the Solicitor of the Association, containing the decision of the Board of Trade:—

Board of Trade (Railway Department),
London, S.W., 6th May, 1891.

Sir,—I am directed by the Board of Trade to say that they have carefully considered the application for a license under Section 23 of the Companies Act, 1867, authorising the Royal British Nurses' Association to register as a limited company without the use of the word "limited."

The Board of Trade have received a large number of communications from bodies of persons whose interest in hospital nursing is unquestionable, and whose experience entitles them to speak with authority, strongly objecting to the issue of a license.

After careful consideration of the objects of the Association, and of the representations made in opposition thereto, the Board of Trade are unable to satisfy themselves that the means which the Association propose to adopt are either adequate to carry out their objects satisfactorily or so free from objection as to warrant the Board of Trade in the issue of a license, and that under these circumstances they are unable to accede to the application.

I am, however, to point out that this refusal in no way precludes the Association from registration as an ordinary joint stock company, under which registration they would enjoy the same powers and be subject to no greater responsibilities than would be the case if they were registered without the word "limited."

I am, &c.,

Henry G. Calcraft.

F. S. Randolph, Esq., 3, Old Serjeants' Inn, W.C.

No doubt nurses will read and ponder, and so save themselves any further trouble and anxiety in the matter.

### Everybody's Opinion.

[*Correspondence on all subjects is invited, but we cannot in any way be responsible for the opinions expressed by our correspondents. No communications can be entertained if the name and address of the correspondent is not given, or unless one side of the paper only be written on.*]

### THE DULNESS OF DOMESTIC SERVICE.

"A. E. S." writes: May another mistress of many years' standing be allowed to say a few words on "The Dulness of Domestic Service"? If servants be dull now, what must they have been thirty years ago, when they were hired to work, and expected to do so? Now they engage the place—ask the mistress more questions than she asks them, want to go shopping two or three times a week, want, at least, every other Sunday off, from 2 until 9; Bank Holidays, regattas, circus, concerts, theatres, and picnics, are all expected, and, if refused, the poor mistress finds all too late, she has nothing but sulks to endure, so might as well have let her servants go. There is little but "eye service," there is no such thing as real interest in the work; high wages, little work, is what they all seek; and now it will be "no work between meals." It is much more usual to find a conscientious mistress than a faithful servant. Servants have the power to make either heaven or the other place of a house. They are simply required to do the work they are hired (or engaged) to do, and if only they'd do so, no mistress would ever wish to refuse any possible pleasure. The poor tired mistress drags herself about, feeling ill and worn out, but catch "Mary" doing so if she's only a small headache. After long years of earnestly trying to be kind and friendly to servants, we are constrained to admit that the less they are treated like friends, and the more like slaves, the better chance of getting served has the mistress. They cannot "bear corn," and if one sticks to justice on one's own side, one has the right to expect work to be done in our way, not theirs.

### Wants and Workers.

[Under this heading, we propose to try for a few weeks whether we can be useful to our readers in making the wants of some known to others who are willing to do what work they can to aid the great cause of curing and cheering the sick.]

*A Gift.*—The Nurses' Library, Children's Hospital, Nottingham, would be very glad to have the copies of The Hospital offered by *A. G.*

*Letters Wanted.*—A Queen's Nurse would be very glad of letters for convalescent homes, and also for a water cushion and some macintosh.—*Poor Parish.*

*Piano Wanted.*—For the Children's Hospital, Nottingham. Surely there is someone in Nottingham or Leicester who having bought a "grand" could spare their "cottage" to cheer the sick children.

*Papers.*—23 Ward, St. Pancras Infirmary, King's Road, Camden, would be glad of the comic and illustrated papers offered by *A. G. T.*

*Ladies' Belt.*—The Shipton District Nursing Association would be glad of the belt offered by *V. H.*

### Notes and Queries.

To Correspondents.—1. Questions or answers may be written on post-cards. 2. Advertisements in disguise are inadmissible. 3. In answering a query please quote the number. 4. A private answer can only be sent in urgent cases, and then a stamped addressed envelope must be enclosed. 5. Every communication must be accompanied by the writer's full name and address, not necessarily for publication. 6. Correspondents are requested to help their fellow nurses by answering such queries as they can.

### Queries.

(15) *Medals.*—How much does it cost to have special silver medals struck for nurses? Will some Matron kindly give me all particulars?—*S. G.*

### Answers.

*Inebriates.*—One of the best homes for ladies and gentlemen is that kept by Dr. James Stewart, "Dunmurry," Sneyd Park, Clifton, Bristol.

*Edina.*—Nurses are taken at 22 years of age at Clayton Hospital, Wakefield; Douglas Hospital, Isle of Man; and at Grimsby Hospital.

(14) *Home for Consumptive.*—Try Hon. Sec., Mildmay Home, Torquay; terms from 7s. a-week.—*Sister.* Apply Ribbsford House, Chapel Park Road, St. Leonards on-Sea.—*E. M.*

*E. R. G.*—You may perhaps secure work at Malta by becoming a Naval Nursing Sister. If you go to New York it must be at your own risk, and on the chance of getting work on landing.

(12) *Smell of Sulphur.*—Fresh air and beating are the only means of getting rid of the smell.

*A. W.*—The Workhouse Infirmary Nursing Association would welcome your services; see advertisements.

### "Into the Haven."

"If I only knew I was going to be with Trudie."

That was the burden now pressing so heavily on my patient; the burden I felt sadly I would give so much to lighten. My patient, Mr. Montague, was master of the fox-hounds, and a fortnight previously, when leading the field, he had come to grief over a stiff bullfinch, with the result of injuries so severe that human skill could do little; only death, which was rapidly approaching, could give relief. No fear of death distressed Mr. Montague; only the fear, Would he be with his wife? "Trudie," he said, had been so good, and he felt so unworthy; would he ever stand by her in the "better land"? Gently I strove to tell him the sweet old story, how "Jesus saves from sin," but though he listened courteously, I felt no words of mine touched him; I was powerless to give him comfort. Seven o'clock at night came, the fire was burning brightly, and the one candle I had was carefully shaded; the surgeon had been, and had told me he did not think that my patient would last the night. I felt he was passing away uncomforted. A knock came at the door, followed almost immediately by the entrance of Baby Harvey, a sweet little lad of three, brought in by his nurse, to wish his father "Good-night."

The nurse left the room, and Harvey climbed on a chair by his father's side, and began talking in his pretty baby-fashion. "Mine fader, is 'oo better? I is tum to say 'dood-night.' Sall I say 'oo my hymn?" and without waiting for an answer the childish voice began, "Jesu, Lover of my soul;" on went Harvey, "Safe into the Haven guide, Oh receive my soul at last." On went the childish voice to the end, and light and comfort had been brought to my patient by his little son—"Trudie's boy." As I bent over the bed I heard the weak voice earnestly repeat, "Safe into the Haven guide, Oh receive my soul at last," and all expression of doubt and distress had left the strong manly face; assurance and hope were written there instead.

At midnight quite quietly and peacefully the end came, and I realised thankfully that the "Mighty Pilot" had by a child's hand guided my patient "Safe into the Haven."

### Presentations.

Dr. Buchley Pogson has been presented with a clock as a wedding present, by the Matron and Nurses of Durham County Hospital.

Mr. Harold West has been presented with a silver match box by the Matron and Nurses of the North Cambridgeshire Cottage Hospital on the occasion of his resignation of the position of Surgeon.

Mr. Willmer Phillips who has for five years held the post of House Surgeon in the Windsor Royal Infirmary, this week resigned his office, having entered into private practice at Southsea. Before his departure from Windsor, a valuable gold watch (having large second-hands for medical pu poses) was presented to him. The cost was defrayed chiefly by the staff of the Institution assisted by some of the managers and by several of the present and former patients.

Thursday evening the 28th May was the occasion of presenting a testimonial to Miss Eliza Olive Attree, who has been a Nurse at the St. Pancras Infirmary for the past twenty years. Dr. McCann, the Medical Superintendent; Mr. Croager, the Steward; Miss Moir, the Matron; and a large number of the staff were present. Dr. McCann referred to the long and efficient service of Nurse Attree, with whom it had been his pleasure to work for a great many years. He stated that the very able manner in which she had performed her duties, and the kindness she had exhibited in attending on the sick poor in the Infirmary, had not only gained from them their highest regard and good feeling, but it had also commanded the esteem and respect of all her fellow officers. Dr. McCann then on behalf of the officers, presented to Miss Attree a handsome marble clock and a leather arm-chair; and at the same time wished her every happiness in the well-earned retirement she is now seeking. The presentation was preceded by a social meeting of officers.

### Princess Louise Augusta's Wedding Present.

A meeting of the Wedding Present Committee was held at 8, King Street, Cheapside, on May 26th, Miss Durham, Miss Pigott, Miss Raynor, and Dr. G. W. Potter attended. It was decided to purchase a copy of Lord Tennyson's works and also a crescent of diamonds, the ladies of the Committee being deputed to make the purchases. Messrs. Macmillan and Co., Lord Tennyson's publishers, most generously offer without charge unbound copies of all the poet's works, beautifully printed on the finest hand-made paper, and these will be richly bound in vellum. Lord Tennyson has promised to write a few lines for the occasion, and to inscribe them with his own hand. This presentation, with the original lines and autograph of the venerable laureate, will undoubtedly be the present of the wedding. The presentation will probably be made in the end of June, and further particulars will be given shortly.

### Where to Go.

The next lecture at the Midwives' Institute and Trained Nurses' Club will be given on Friday evening, June 12th, at a quarter to eight, by Mr. Malcolm Morris, F.R.C.S.E., Surgeon to the Skin Department, St. Mary's Hospital, Paddington—subject: "The Management of the Skin in Health and Disease." A few tickets to non-members 6d. each, for which early application must be made to the Secretary.

The cold weather seems to have departed at last, and plans can be made for a long "day off" in the open air. The s.s. *Lord of the Isles* leaves the Old Swan Pier, London Bridge, every morning except Friday, for a trip to Ipswich and back. The *Cardinal Wolsey* leaves the same pier every day, including Sundays, at ten a.m. for Hampton Court and back.

The coaching season has commenced, and coaches are running daily to Virginia Water, through Epping Forest and to Windsor and elsewhere.

Polytechnic excursions to Switzerland (16 days tour for £7), to Scotland (£2 10s. for 7 days), and to Norway £8 10s. for 13 days) are being arranged. Particulars can be had on application to the Secretary, Polytechnic, Regent Street, W.

### Amusements and Relaxation.

SPECIAL NOTICE TO CORRESPONDENTS.

**Second Quarterly Word Competition commenced April 4th, ends June 27th, 1891.**

Competitors can enter for *all* quarterly competitions, but no competitor can take more than *one first prize* or *two* prizes of any kind during the year.

The words for dissection for this, the TENTH week of the quarter, being

"SEASIDE."

| Names. | May 28th. | Totals. | Names. | May 28th. | Totals. |
|---|---|---|---|---|---|
| Christie | 20 | 350 | Ivanhoe | 20 | 293 |
| Patience | — | 214 | Weta | — | — |
| Agamemnon | 19 | 352 | Lady Betty | — | — |
| Hope | 20 | 352 | Mortal | — | 76 |
| Reldas | 20 | 354 | Little Eliza | — | — |
| Lightowlers | 20 | 326 | Dove | — | 95 |
| Nurse J. S. | 19 | 295 | Ladybird | — | — |
| Qu'appelle | — | — | Psyche | 19 | 311 |
| Jenny Wren | 19 | 279 | Ugug | — | — |
| Wyameris | 19 | 354 | Harrie | 16 | 125 |
| Paignton | 19 | 294 | Grannie | 20 | 291 |
| Theta | — | 206 | Eale | — | 169 |
| Success | — | 17 | Grimalkin | — | 53 |
| Tired | — | 136 | Nurse G. P. | 20 | 88 |
| M. G. | — | 188 | | | |

### Notice to Correspondents.

N.B.—All letters referring to this page which do not arrive at 140, Strand, London, W.C., by the *first post on Thursdays*, and are not addressed PRIZE EDITOR, will in future be disqualified and disregarded.

## Figures and Tables

**Figure f1:**